# Systematic review of bidirectional interaction between gut microbiome, miRNAs, and human pathologies

**DOI:** 10.3389/fmicb.2025.1540943

**Published:** 2025-02-05

**Authors:** Lorenzo Drago, Luigi Regenburgh De La Motte, Loredana Deflorio, Delia Francesca Sansico, Michela Salvatici, Emanuele Micaglio, Manuele Biazzo, Fabiana Giarritiello

**Affiliations:** 1 UOC Laboratory of Clinical Medicine with Specialized Areas, IRCCS MultiMedica, Milan, Italy; 2 Clinical Microbiology and Microbiome Laboratory, Department of Biomedical Sciences for Health, University of Milan, Milan, Italy; 3 Genetic Consultant, IRCCS Multimedica, Milan, Italy; 4 The BioArte Ltd., Life Science Park, San Gwann, Malta; 5 Department of Medicine and Health Sciences “V. Tiberio”, University of Molise, Campobasso, Italy

**Keywords:** miRNA, gut microbioma, inflammatory bowel disease (IBD), Crohn’s disease (CD), Alzheimer’s disease (AD), major depressive disorder (MDD), cardiovascular disease

## Abstract

MicroRNAs (miRNAs) and the gut microbiome are key regulators of human health, with emerging evidence highlighting their complex, bidirectional interactions in chronic diseases. miRNAs, influence gene expression and can modulate the composition and function of the gut microbiome, impacting metabolic and immune processes. Conversely, the microbiome can affect host miRNA expression, influencing inflammatory pathways and disease susceptibility. This systematic review examines recent studies (2020–2024) focusing exclusively on human subjects, selected through rigorous inclusion and exclusion criteria. Studies were included if they investigated the interaction between miRNAs and the gut microbiome in the context of gastrointestinal diseases, obesity, autoimmune diseases, cognitive and neurodegenerative disorders, and autism. *In vitro*, *in vivo* and in silico analyses were excluded to ensure a strong translational focus on human pathophysiology. Notably, miRNAs, stable and abundant in patients, are emerging as promising biomarkers of microbiome-driven inflammation. This systematic review provides an overview of miRNAs, their regulatory effects on bacterial strains, and their associations with specific diseases. It also explores therapeutic advances and the potential of miRNA-based therapies to restore microbial balance and reduce inflammation.

## 1 Introduction

MicroRNAs (miRNAs), short non-coding RNAs (~22 nucleotides), are post-transcriptional regulators of gene expression. They function by binding to the 3′ untranslated region (UTR) of mRNAs resulting in either mRNA degradation or translational repression. This refined regulation influences a wide range of biological processes, including cell differentiation, proliferation, and apoptosis (Winter et al., 2009). Recent studies have shown that miRNAs and the gut microbiome interact within intestinal epithelial cells, influencing critical signaling pathways and regulating gene expression (Dong et al., 2019; Li et al., 2010). The disruption of the symbiotic relationship between the gut microbiome and the host has been implicated in numerous human pathologies, including cancer, autoimmune disorders, type 2 diabetes mellitus (T2DM), cardiovascular diseases (Iliopoulos et al., 2009; Ha, 2011). Genetic and epigenetic alterations of specific miRNAs have been linked to disease development, and in recent years, miRNA-based therapies have shown potential for treating a variety of illnesses (Hydbring and Badalian-Very, 2013). Furthermore, growing evidence highlights the crucial role of miRNAs in regulating host responses to pathogenic infections (Maudet et al., 2014; Riaz Rajoka et al., 2018). The gut microbiome, a complex ecosystem comprising trillions of bacteria and other microorganisms in the digestive tract, includes six major anaerobic bacterial groups: *Prevotella*, the *Bacteroides fragilis* group, the *Atopobium* cluster, *Bifidobacterium*, the *Clostridium leptum* subgroup and the *Clostridium coccoides* group. Additionally, five potential pathogenic clusters, such as *Pseudomonas*, *Staphylococcus*, *Enterococcus*, *Enterobacteriaceae* and *Clostridium perfringens*, are part of this intricate community. The microbiome may also contains eight types of *Lactobacillus*, including the *L. sakei* group, the *L. reuteri* group, the *L. ruminis* group, the *L. plantarum* group, the *L. casei* group, *L. fermentum*, *L. brevis* and the *L. gasseri* group (Hu et al., 2013; Boni et al., 2010). Alterations in this microbial balance, known as dysbiosis, have been implicated also in neurodegenerative disorders, including Alzheimer’s disease, major depressive disorder and autism (Durack and Lynch, 2019). In gastrointestinal cancers, including gastric and colorectal cancer, miRNAs and the gut microbiota interact to influence tumorigenesis, immune evasion, and inflammation. miRNAs can act as oncogenes or tumor suppressors, modulating the expression of genes involved in cancer progression. Dysbiosis further exacerbates these processes by generating pro-inflammatory metabolites and altering local immune responses (Lee et al., 2010; Yau et al., 2019; Ji et al., 2018; Shen et al., 2022). Beyond cancer, dysbiosis has also been associated with IBS and fecal constipation, where microbial imbalances disrupt gut motility and barrier function. The miRNA-microbiome axis is also critical in metabolic and systemic disorders (Viennois et al., 2017). In T2DM and obesity, dysregulated miRNA expression influences glucose homeostasis and adipocyte differentiation, while alterations in the microbiome contribute to low-grade systemic inflammation and insulin resistance (Liu et al., 2016). Neurodegenerative and cognitive disorders, including Alzheimer’s disease, mild cognitive impairment, major depressive disorder, and autism spectrum disorder, represent another frontier in understanding the miRNA-microbiome-disease axis. Microbial dysbiosis and altered miRNA expression can disrupt the gut-brain axis, leading to neuroinflammation and cognitive decline. Emerging evidence suggests that microbiome-targeting interventions may hold promise for these conditions. This would help in understanding the mechanistic pathways through which miRNAs and the microbiome influence disease progression. Additionally, investigating their roles in diagnostics could reveal miRNA profiles or microbiome signatures that could serve as non-invasive biomarkers for early disease detection. Moreover, therapeutic strategies targeting miRNAs or modulating the gut microbiome could offer new avenues for treating a range of diseases.

## 2 Materials and methods

**Figure 1:**
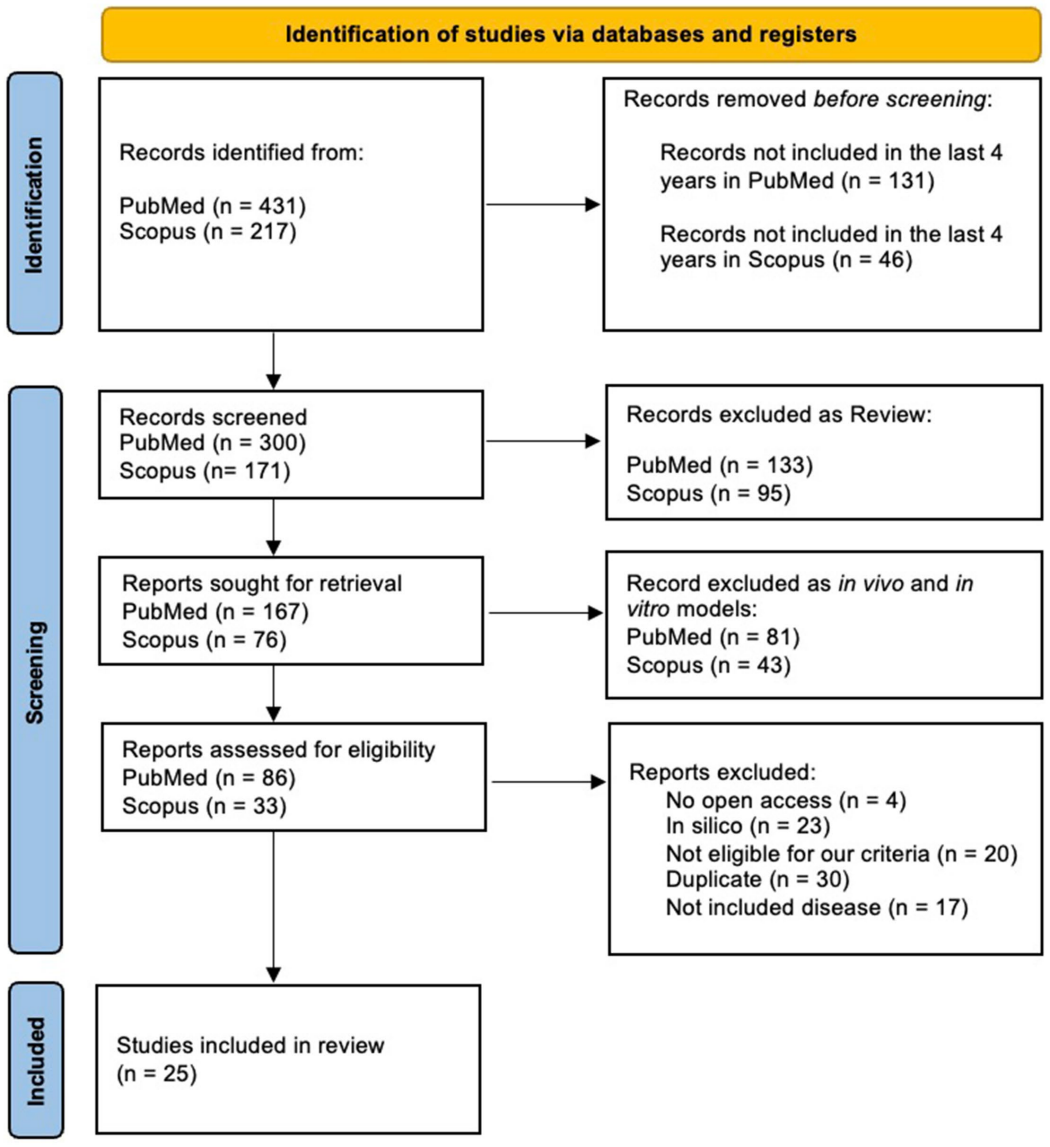
PRISMA flowchart of articles identification and selection strategy.

In this systematic review, we aimed to examine the interaction between miRNAs and the intestinal microbiome, focusing on their bidirectional influence and roles in the pathogenesis of human diseases. To ensure a comprehensive and unbiased approach, we conducted a systematic search using both PubMed and Scopus databases. We conducted independent searches in PubMed and Scopus databases using the following query: (microRNA OR miRNA) AND (gut microbiota OR intestinal microbiome) AND (interaction OR association) NOT review NOT *in vivo* NOT *in vitro*. The search ware restricted to articles published between 2020 and 2024 to ensure the inclusion of the most recent findings. The search in PubMed yielded 431 records, while 217 articles were identified in Scopus. After applying filters to exclude reviews, *in vivo*, and in vitro studies—given the variability and bioavailability of the microbiome in human subjects—the datasets were reduced to 300 and 171 articles, respectively. During an initial screening phase, 133 PubMed and 95 Scopus articles classified as reviews were excluded, as were 81 PubMed and 43 Scopus articles focused on *in vitro* or *in vivo* models. This left 167 PubMed articles and 76 Scopus articles for further evaluation. In the eligibility assessment phase, 86 articles from PubMed and 33 from Scopus were reviewed in detail. Exclusions were made for lack of open access (4 articles), reliance solely on *in silico* analyses (23 articles), failure to meet inclusion criteria (20 articles), duplication across databases (30 articles), or addressing diseases outside the scope of this review (17 articles). Ultimately, 25 studies were included in the final analysis, all focusing on patients and exploring the miRNA-microbiome axis in the context of disease mechanisms. The selection process followed the Preferred Reporting Items for Systematic Reviews and Meta-Analyses (PRISMA) guidelines (Page et al., 2021), as illustrated in the flowchart in Figure 1. This rigorous methodology ensured the inclusion of studies focusing exclusively on human subjects, addressing the mechanisms by which miRNAs modulate the intestinal microbiome and vice versa. This chart provides a structured summary of each criterion applied, ensuring transparency and clarity in our methodological approach.

## 3 Interactions between gut microbiome and miRNAs in gastrointestinal diseases

MiRNAs have been shown to play a critical role in a wide range of physiological and pathological processes (Ferruelo et al., 2018). Accumulating evidence indicates that imbalances in the gut microbiota and alterations in miRNA expression levels are closely linked to the onset and progression of various human diseases. Both factors can act as triggers for diverse disorders and have been identified as promising biomarkers, particularly in conditions such as type 2 diabetes mellitus (T2DM; Teng et al., 2018; Williams et al., 2017). Several studies suggest that the gut microbiota interacts with miRNAs in regulating host physiology and immune processes. MiRNAs can mediate the crosstalk between gut microbiota and the host by modulating immune responses and influencing microbial composition. Notably, miRNAs are capable of directly targeting bacterial genes, thereby regulating bacterial communities and acting as markers for microbial fluctuations in intestinal pathologies (Moloney et al., 2018). This bidirectional relationship highlights the dynamic interplay between host miRNA networks and the gut microbiome, particularly in the context of chronic diseases. While the role of miRNAs in aberrant intestinal inflammation and disease recurrence, such as in Crohn’s disease (CD), remains poorly defined, emerging evidence underscores their ability to orchestrate immune and microbial dynamics. For example, through a holistic analysis of ileal microbiota composition, circulating free fatty acids (FFAs), and miRNAs, researchers have begun to unravel the mutual dialog between microbial and inflammatory factors in CD, comparing healthy and pathological tissues. This section explores how miRNAs, through their upregulation or downregulation, modulate immune responses and microbiome composition, ultimately shaping disease progression. A comprehensive overview of the specific miRNAs involved, their regulatory roles, and their associations with particular diseases and microbial signatures is presented in Table 1.

**Table 1:** Comprehensive overview of dysregulated microbiome and miRNA interactions in disease states.

Disease/condition	Dysregulated microbiome	miRNAs	Function
Crohn’s Disease (CD)	↑ *Escherichia coli,* ↑ *Shigella sonnei*, ↓ *Roseburia intestinalis* ↓ *Defluviitalea raffinosedens*	↓ miR-146a ↓ miR-21 ↑ miR-223-3p	Regulates immune response (Tregs), promotes dysbiosis, and reduces SCFA production.
Gastric Cancer (GC)	↑ *Lacticaseibacillus* ↓ *Haemophilus,* ↓ *Campylobacter*	↓ miR-124-3 ↓ TWIST1	Silences tumor suppressor pathways (drives inflammation through cytokines such as TNF-α and CXCL9), promotes epithelial-mesenchymal transition.
Colorectal Cancer (CRC)	↑ *Fusobacterium nucleatum*	↓ miR-1322	Promotes tumor growth and immune suppression via the CCL20 axis.
Functional constipation	↑ *Oscillibacter* ↑ *Escherichia-Shigella* ↓ *Megamonas*	↓ miR-205-5p ↓ miR-215-5p ↑ miR- 378c	Dysbiosis and miRNA alterations together disrupt gastrointestinal motility and contribute to symptom severity.
Irritable bowel syndrome	↓ *Bacteroidetes* ↑ *Proteobacteria* ↑ *Lachnospiraceae* ↑ *Succinivibrionaceae UCG-001* ↓ *Marvinbryantia* ↓ *Alistipes*	↓ miR-16	miRNA regulates tight junction proteins and inhibits inflammatory pathways such as TLR4/NF-κB. Its downregulation may exacerbate gut permeability and inflammation.
Chronic HBV Infection	↓ *Bacteroides fragilis,* ↓ *Lactobacillus* ↓ *Bifidobacterium*	↑ miR-192-5p ↔ miR-122-5p	Impacts glucose metabolism. This regulatory role link to insulin resistance and impaired glucose homeostasis, highlighting its potential role of T2DM’s target.
Obesity	↑ *Bacteroides eggerthii* ↔ *Barnesiella intestinihominis* ↑ *Dorea longicatena* ↔ *Clostridia* ↔ *Burkholderia*	↔ miR-130b-3p ↔ miR-185-5p ↑ miR-21-5p ↔ miR-30 ↔ miR-194	Modulate host metabolism but also shape the biosynthetic landscape of the microbiome, with potential implications for weight regulation.
Systemic Lupus Erythematosus (SLE)	↑ *Collinsella,* ↑*Bifidobacterium* ↑ *Lactobacillus* ↓ *Odoribacter* ↓ *Lachnospiraceae NK4A136*	↓ miR-223-3p ↔ miR-146a-5p ↔ miR-155-5p	Immune modulation, correlates with inflammatory markers, regulates TLR4 pathway.
Hashimoto’s Thyroiditis (HT)	↑ *Peptostreptococcus* ↑ *Klebsiella* ↑ *Streptococcus* ↓ *Barnesiella* ↓ *Catonella*	↑ miR-548aq-3p ↑ miR-374a-5p	Promote inflammatory pathways and reduce short-chain fatty acid (SCFA) production, exacerbating immune dysfunction and thyroid inflammation.
Diabetes-Associated Cognitive Dysfunction (DACD)	↑ *Eubacterium coprostanoligenes* ↑ *Enterorhabdus* ↑ *E. coprostanoligenes* ↑ *Clostridium methylpentosum* ↑ *Acidaminococcus* ↑ *Ruminococcaceae* ↑ *Lacillobacteriaceae* ↓ *Anaeroglobus* ↓ *Lactobacillales* ↓ *Bradyrhizobium* ↓ *Porphyromonas* ↓ *Erysipelotrichaceae UCG-006* ↓ *Lawsonella*	↑ miR-142-5p ↑ miR-107 ↑miR-155-5p ↑ miR-144-3p	Promotes inflammation, linked to cognitive dysfunction.
Autism spectrum disorder (ASD)	↓ *Ruminococcus* ↓ *Akkermansia* ↓ *Bifidobacterium* ↑ *Desulfovibrio* ↑ *Coprococcus* ↑ *Alistipes* ↑ *Sutterella*	↑ miR-182-5p ↑ miR-681 ↑ miR-657 ↑ miR-2110 ↓ miR-192-5p	Regulation gut permeability and inflammation.
Major Depressive Disorder (MDD)	↑ *Bacteroide* ↑ *Streptococcus* ↑ *Klebsiella* ↓ *Dialister* ↓ *Bifidobacterium* ↓ *Faecalibacterium* ↓ *Roseburia.*	↑ miR-3144-3p ↑ miR-579-3p ↔ miR-1976 ↔ miR-1276	Regulates microbial colonization, affects emotional pathways.
Mild Cognitive Impairment (MCI)	↑ *Proteobacteria* ↑ *Gammaproteobacteria* ↓ *Faecalibacterium* ↓ *unidentified Ruminococcaceae* ↓ *Alistipes* ↓ *Rikenellaceae*	↑ miR-144-3p ↓ let-7 g-5p ↓ miR-107 ↓ miR-186-3p	Modulation of genes expression under pathways affecting microbial colonization and cognitive health.

This table provides a comprehensive summary of the associations between dysregulated microbial signatures and miRNA expression across a range of diseases. The interactions detailed underscore the role of microbial imbalances in modulating miRNA expression, thereby influencing disease pathogenesis through mechanisms such as immune modulation, inflammatory processes, and disruptions in metabolic pathways. The upward arrow (↑) denotes upregulation, the downward arrow (↓) signifies downregulation, and the bidirectional arrow (↔) represents a correlation warranting further investigation.

### 3.1 Crohn’s disease

The intricate relationship between microRNAs (miRNAs) and the gut microbiome has become a focal point in understanding the mechanisms of gastrointestinal diseases, particularly in inflammatory conditions such as Crohn’s disease. In Crohn’s disease (CD), the intestinal microbiota exhibits distinctive microbial signatures that are closely linked to the regulation of miRNAs. Recent studies have identified seven key bacteria that play a crucial role in the pathogenesis of CD, including *Defluviitalea raffinosedens, Thermotalea metallivorans, Roseburia intestinalis, Dorea* sp. *AGR2135, Escherichia coli, Shigella sonnei, and Salmonella enterica* (Lv et al., 2024). The composition of these bacteria is modulated by the expression of miRNAs, which in turn influence intestinal inflammation and disease progression. For instance, down-regulated miRNAs in CD, such as miR-146a and miR-21, are essential in regulating the immune response and the function of regulatory T cells (Tregs; Mirzakhani et al., 2020; Cobb et al., 2006; Harvey and Bradshaw, 1980; Rossi et al., 2011; Lu et al., 2010). The down-regulation of miR-146a compromises immune tolerance, promoting inflammation and altering bacterial composition by reducing beneficial bacteria such as *R. intestinalis* (a producer of SCFAs) and increasing pathogens like *E. coli* (Darfeuille-Michaud et al., 2004; Fang et al., 2018; Lloyd-Price et al., 2019). Similarly, miR-21, another down-regulated miRNA, affects immune response and the microbiome as its reduction leads to T cell hyperactivation, contributing to an inflammatory environment that promotes the growth of pathogens such as *S. sonnei* and *S. enterica*. In contrast, up-regulated miRNAs such as miR-223-3p, a pro-inflammatory miRNA, are associated with the activation of the IL-23 inflammatory cascade and the regulation of bacterial genes involved in intestinal barrier permeability, such as claudin-8, whose alteration promotes pathogen growth. The up-regulation of miR-223-3p thus contributes to a feedback loop that promotes dysbiosis, where pathogenic bacteria dominate the intestinal microbiota, while protective bacteria such as *D. raffinosedens* are inhibited (Lv et al., 2024; Ambrozkiewicz et al., 2020). miRNA dysregulation contributes to intestinal inflammation by altering immune responses and bacterial composition, creating a feedback cycle that worsens the CD progression.

### 3.2 Gastrointestinal diseases

The pathogenesis of gastrointestinal diseases, including gastric cancer (GC), colorectal cancer (CRC), irritable bowel syndrome (IBS), and functional constipation (FC), is intricately linked to microbial dysbiosis and miRNA regulation.

In gastric cancer, microbial imbalances such as the enrichment of *Lacticaseibacillus* and reduction of *Haemophilus* and *Campylobacter* create a pro-inflammatory environment that promotes epigenetic alterations, including the hypermethylation and silencing of miRNAs like miR-124-3 and TWIST1 negative related to *Helicobacter pylori*-negative GC (Ushijima et al., 2006; Yang et al., 2018). These changes disrupt tumor-suppressor pathways and enhance epithelial-to-mesenchymal transition, contributing to tumor progression. Similarly, fungal dysbiosis in GC, including enrichment of *Apiotrichum* and *Cutaneotrichosporon*, drives inflammation through cytokines such as TNF-*α* and CXCL9, which suppress tumor-suppressive miRNAs and promote oncogenic pathways(Kim et al., 2024; Kim et al., 2021; Yang et al., 2022).

In colorectal cancer, *Fusobacterium nucleatum* plays a critical role in tumor progression by modulating the miR-1322/CCL20 axis. This bacterium suppresses miR-1322 via the NF-κB signaling pathway, increasing the expression of CCL20 (Kostic et al., 2013), promoting immune cell infiltration, and polarizing macrophages toward a pro-tumor phenotype. Such interactions highlight the dynamic relationship between microbial dysbiosis and miRNA modulation in creating a pro-tumor microenvironment in gastrointestinal cancers (Xue et al., 2018; Xu et al., 2021; Gur et al., 2019).

In functional constipation, microbial shifts such as increases in *Oscillibacter* and *Escherichia*-*Shigella*, coupled with reductions in *Megamonas*, are accompanied by changes in miRNA expression. Downregulation of miRNAs like miR-205-5p and miR-215-5p correlates with impaired colonic motility and increased inflammation, while upregulation of miR-378c influences epithelial barrier function and cytokine signaling. These findings suggest that dysbiosis and miRNA alterations together disrupt gastrointestinal motility and contribute to symptom severity (Yao et al., 2023).

In irritable bowel syndrome, microbial dysbiosis is similarly central to disease mechanisms. A starch- and sucrose-reduced diet has been shown to shift microbial populations, reducing *Bacteroidetes* and increasing *Proteobacteria*, which correlates with symptom improvement. At the genus level, increases in *Lachnospiraceae*, *UCG-001* and decreases in *Marvinbryantia* are associated with dietary intervention (Nilholm et al., 2022). Furthermore, *Blastocystis* infection in IBS patients has been linked to reductions in beneficial bacteria such as *Alistipes*, alongside altered miRNA expression. Notably, miR-16, a key modulator of gut barrier integrity, is downregulated in *Blastocystis*-positive IBS patients. This miRNA regulates tight junction proteins and inhibits inflammatory pathways such as TLR4/NF-κB. Its downregulation may exacerbate gut permeability and inflammation, contributing to IBS symptomatology (Olyaiee et al., 2023). These findings underscore the interplay between microbial diversity, miRNA regulation, and gastrointestinal health.

### 3.3 Hepatitis B virus and miRNA regulation in diabetes mellitus

Hepatitis B virus (HBV) infection has been shown to contribute to intestinal microbiota imbalance, playing a potential role in the development of diabetes mellitus (DM). Intestinal dysbiosis in HBV is characterized by a reduction in beneficial bacterial genera such as *Lactobacillus, Bifidobacterium*, and *Bacteroides fragilis*. This imbalance correlates with elevated levels of miR-192-5p, which suppresses the synthesis of glucagon-like peptide-1 (GLP-1) by binding to its 3′ untranslated region (UTR), thereby impairing glucose metabolism and exacerbating diabetic conditions. Additionally, miR-192 induced GLP-1 suppression creates a feedback loop that perpetuates gut microbiota imbalance and systemic inflammation, further accelerating disease progression (Liu, 2021; Pan et al., 2018). These bacteria, such as *Bacteroides uniformis*, are negatively correlated with circulating miR-122-5p levels, suggesting a reciprocal regulatory mechanism. Elevated miR-122-5p impacts glucose metabolism by targeting genes involved in gluconeogenesis and glycolysis pathways, such as glucose-6-phosphatase (G6PC), phosphoenolpyruvate carboxykinase (PEPCK), and glucose transporter type 2 (GLUT2). This regulatory role links miR-122-5p to insulin resistance and impaired glucose homeostasis, highlighting its role as a potential therapeutic target for T2DM (Al-Kafaji et al., 2015). Moreover, bioinformatics analyses reveal that *Bacteroides uniformis* and *Phascolarctobacterium faecium* may enhance insulin sensitivity and reduce inflammation through their metabolic pathways, including butyrate production and polyphenol metabolism (Pan et al., 2018). Their decline in T2DM patients underscores the importance of maintaining microbial diversity to counteract the pathological effects of miR-122-5p dysregulation. These findings underline the complex interplay between HBV-induced dysbiosis, miRNA regulation, and metabolic dysfunction. While miR-192 contributes to GLP-1 suppression and systemic inflammation, miR-122-5p modulates key metabolic pathways in glucose homeostasis. This bidirectional relationship between gut microbiota and miRNA expression highlights novel avenues for therapeutic interventions aimed at restoring microbiota balance and targeting miRNA-mediated regulatory networks (Li et al., 2020).

## 4 Obesity

Obesity, a global epidemic resulting from chronic energy imbalance, is increasingly linked to complex interactions between the gut microbiota and host miRNAs. Recent studies have illuminated the role of miRNAs in regulating metabolic pathways and the crosstalk between microbial composition and host gene expression. In particular, miRNAs have emerged as key mediators in shaping microbial communities and modulating host metabolic responses. The expression profiles of miR-130b-3p, miR-185-5p, and miR-21-5p, were found to differ significantly between obese and healthy individuals. These miRNAs demonstrated correlations with bacterial species such as *Bacteroides eggerthii*, *Barnesiella intestinihominis*, and *Dorea longicatena*, which are implicated in metabolic regulation. Notably, miR-21-5p, which targets pathways involved in insulin signaling and glycerolipid metabolism, was upregulated in obesity, potentially contributing to systemic inflammation and metabolic dysfunction. Furthermore, *Bacteroides eggerthii*, enriched in obese subjects, correlated with altered miRNA expression, highlighting the bidirectional communication between miRNAs and microbial taxa (Assmann et al., 2020). Another study focused on infants revealed associations between stool miRNAs and microbial activity during early growth, shedding light on mechanisms that may influence long-term obesity risk. The miR-30 family and miR-194, known to regulate adipocyte differentiation and inflammation, were linked to microbial taxa such as *Clostridia* and *Burkholderia*. These findings suggest that miRNAs not only modulate host metabolism but also shape the biosynthetic landscape of the microbiome, with potential implications for weight regulation. Importantly, the transcriptional activity of these microbes correlated with infant weight-for-length z-scores, reinforcing the role of miRNAs in mediating host-microbiota interactions during critical developmental windows (Carney et al., 2021). Together, these studies underscore the intricate relationship between miRNAs and the gut microbiome in obesity. By regulating metabolic and inflammatory pathways, miRNAs act as crucial intermediaries in the host-microbe dialog. The dynamic interplay between dysbiosis and miRNA dysregulation provides insights into potential therapeutic targets aimed at restoring metabolic balance and preventing obesity-related complications.

## 5 Autoimmune diseases

An important development has been the growing understanding of the relationship between the gut microbiome, miRNAs, and the progression of autoimmune diseases. Systemic Lupus Erythematosus (SLE) and Hashimoto’s Thyroiditis (HT) are two autoimmune conditions where the interplay between gut dysbiosis, miRNAs, and immune responses has been extensively studied. SLE, a chronic autoimmune and multisystem inflammatory disease, demonstrates a complex pathogenesis influenced by genetic and environmental factors (Tsokos, 2011; Tsokos, 2020). Similarly, HT is characterized by an immune-mediated attack on the thyroid gland, leading to chronic inflammation and eventual hypothyroidism. In both diseases, the gut microbiome and miRNAs emerge as key players in modulating immune tolerance and inflammation. In SLE, intestinal dysbiosis impacts the phenotype and function of T cells, B cells, and plasmacytoid dendritic cells (pDCs), promoting immune tolerance breakdown and exacerbating the disease (Chang and Choi, 2023; Monticolo et al., 2023). Patients exhibit altered gut microbiota, including an overrepresentation of gram-positive bacteria such as *Collinsella, Bifidobacterium, Lactobacillus, Streptococcus,* and *Marvinbryantia*, and a reduction in anti-inflammatory bacteria like *Odoribacter* and the *Lachnospiraceae NK4A136 group* (Chen et al., 2021). miRNAs such as miR-146a-5p, miR-155-5p, and miR-223-3p regulate immune pathways, including TLR4, and correlate with inflammatory markers and microbial changes (Chi et al., 2021; Penas-Steinhardt et al., 2012; Dong et al., 2019). Notably, hsa-miR-223–3p is downregulated, highlighting its potential as a non-invasive biomarker for SLE progression (Abdul-Maksoud et al., 2021). In HT, dysbiosis is characterized by an increased abundance of *Peptostreptococcus*, *Klebsiella*, and *Streptococcus*, alongside reduced levels of butyrate-producing bacteria such as *Barnesiella* and *Catonella*. These shifts promote inflammatory pathways and reduce short-chain fatty acid (SCFA) production, exacerbating immune dysfunction and thyroid inflammation. Dysregulated miRNAs, such as hsa-miR-548aq-3p and hsa-miR-374a-5p, target genes involved in immune-inflammatory pathways, while specific single nucleotide polymorphisms (SNPs) influence microbial diversity and immune interactions (Li et al., 2024). Integrative analyses have revealed novel microbial signatures and miRNA-mRNA networks driving HT pathophysiology, offering potential targets for diagnostics and therapeutics.

## 6 Cognitive disorders

Recent studies have highlighted the crucial role of gut microbiome and miRNAs in cognitive dysfunction and neurodegenerative diseases such as Type 2 diabetes (T2DM), diabetes-associated cognitive dysfunction (DACD), major depressive disorder (MDD), mild cognitive impairment (MCI), Alzheimer’s disease (AD) and Autism spectrum disorder (ASD). The gut microbiome influences brain function and cognition through the microbiome-gut-brain axis, which is a key area of research.

### 6.1 Diabetes-associated cognitive dysfunction

In patients with diabetes-associated cognitive dysfunction (DACD), changes in gut microbiome are closely linked to cognitive decline. These patients exhibit distinct alterations in their gut microbiome. Notably, *Eubacterium coprostanoligenes* group and *Enterorhabdus* were overexpressed, both positively associated with the upregulated miR-142-5p, suggesting a role in promoting inflammation. Additionally, *E. coprostanoligenes* group was associated with the upregulated miR-107. Other overexpressed bacterial families included *Clostridium methylpentosum* group*, Acidaminococcus, Ruminococcaceae*, and *Lacillobacteriaceae.* In contrast, several families were reduced in DACD, including *Anaeroglobus*, which was negatively correlated with the downregulated miR-155-5p, and *Lactobacillales*, which showed a positive correlation with the upregulated miR-144-3p, potentially indicating protective effects (Liu et al., 2020). Hongying et al., found also other downregulated families including *Bradyrhizobium, Porphyromonas, Erysipelotrichaceae UCG-006,* and *Lawsonella* (Huang et al., 2023). These findings highlight a complex interaction between gut microbiota and miRNAs, with key players like miR-142-5p and miR-155-5p potentially mediating inflammatory processes linked to cognitive dysfunction. Further studies are required to elucidate the mechanistic pathways underlying these interactions and their contribution to cognitive impairment in DACD (Zhang et al., 2021; Bi et al., 2021).

Diet also plays a significant role in shaping gut microbiome composition. A “Western diet” high in fat and low in fiber, promotes the growth of harmful bacteria such as *Proteobacteria* and *Enterobacteriaceae*, while a plant-based diet encourages beneficial bacteria (Turpin et al., 2016; Beam et al., 2021). Moreover, increased intake of n-3 polyunsaturated fatty acids (PUFAs) has been shown to reduce the risk of T2DM and cognitive decline, with studies demonstrating a protective effect of n-3 PUFAs against cognitive impairment in T2DM patients (Nguyen et al., 2022; Li et al., 2022).

### 6.2 Autism spectrum disorder

Autism spectrum disorder (ASD) is a complex neurodevelopmental condition characterized by deficits in communication, social interaction, and repetitive behaviors, alongside frequent comorbidities such as gastrointestinal dysfunction and immune dysregulation. Recent research has identified dysregulated miRNAs and gut microbiota imbalances as central players in ASD pathophysiology, underscoring their intricate interplay within the gut-brain axis. Individuals with ASD exhibit significant alterations in miRNA profiles, including upregulated hsa-miR-182-5p, has-miR-681, has-miR-657 and hsa-miR-2110, which are associated with increased intestinal inflammation and disruptions to gut microbiota composition (Chiappori et al., 2022). Notably, hsa-miR-192-5p, a miRNA involved in maintaining intestinal barrier integrity, is significantly downregulated in ASD, further exacerbating gut permeability and inflammation. ASD individuals show a reduced abundance of beneficial bacteria such as *Ruminococcus*, *Akkermansia* and *Bifidobacterium* coupled with elevated levels of pro-inflammatory taxa like *Desulfovibrio*, *Coprococcus*, *Alistipes*, and *Sutterella*. These microbial imbalances negatively impact short-chain fatty acid (SCFA) production and gut homeostasis. In contrast, neurotypical controls exhibit a balanced gut microbiota that supports the regulation of miRNAs, maintaining intestinal homeostasis and neuroprotection. The bidirectional relationship between miRNAs and the microbiota not only highlights the gut’s role in influencing brain function but also presents opportunities for therapeutic intervention. For instance, dietary strategies such as ketogenic diets have shown promise in modulating both miRNA expression and microbial diversity (Allan et al., 2024; Li et al., 2021; Rawat et al., 2021; Lee et al., 2018). By enhancing the abundance of butyrate-producing bacteria and normalizing inflammatory profiles, such interventions demonstrate potential for mitigating ASD symptoms. Understanding these mechanisms paves the way for personalized therapies targeting the miRNA-microbiota axis to improve health outcomes in ASD.

### 6.3 Major depressive disorder

In the context of major depressive disorder (MDD), which often presents with cognitive symptoms, the relationship between gut microbiome and microRNAs has become a subject of increasing interest. Individuals with depressive disorders frequently report gastrointestinal dysfunction and disordered bowel habits, suggesting a robust correlation between gut microbiome and brain health. Recent studies investigating the causal effects of gut microbiome on mental health have indicated that interactions between the microbiome and host miRNAs play a crucial role in regulating emotional and cognitive processes (Chiappori et al., 2022; Tarallo et al., 2022; Han et al., 2022). Hui-Mei et al. showed that in patients with MDD, significant changes in the microbiota were found, including increased levels of *Bacteroide, Streptococcus*, and *Klebsiella*, along with reduced levels of *Dialister*, *Bifidobacterium*, *Faecalibacterium*, and *Roseburia*. These changes are accompanied by alterations in the *Bacteroidetes* and *Actinobacteria* phyla, with reduced microbial diversity correlating with systemic inflammation Functional analysis of the gut microbiota in MDD patients indicates an enrichment in lipopolysaccharide (LPS) biosynthesis and tryptophan metabolism pathways, both of which are linked to inflammatory responses and serotonergic dysfunction. In contrast, healthy controls exhibit a predominance of anti-inflammatory bacteria like *Faecalibacterium* and *Roseburia*, which promote gut-brain axis homeostasis. Concomitantly, several fecal miRNAs, like miR-1276, miR-3144-3p, miR-1976, miR-579-3p and miR-124-3p were shown to be differentially in MDD patients. For example, miR-1976 shows a positive association with *Bifidobacterium*, targeting long-term potentiation pathways, whereas miR-1276 exhibits a negative association with *Collinsella* (Chen et al., 2022). miR-124-3p regulate genes involved in immune responses and serotonergic signaling, further highlighting the intricate interplay between miRNAs and gut microbiota. This bidirectional interaction may indicate that host-derived miRNAs modulate microbial compositions, while microbiota metabolites regulate miRNA expression, collectively impacting gut integrity, inflammation, and brain function. Adding a genetic dimension, specific single nucleotide polymorphisms (SNPs) have been identified as key factors influencing gut microbiota diversity in MDD. These genetic predispositions exacerbate microbial imbalances, further impairing metabolic and immune pathways associated with the condition. The integrative framework of host genetics, gut microbiota, and miRNAs underscores a complex network driving MDD pathophysiology, paving the way for targeted interventions. Therapeutic approaches focusing on restoring gut microbiota diversity and targeting miRNA dysregulation hold promise for alleviating MDD symptoms. Strategies may include probiotic supplementation with *Faecalibacterium* and *Roseburia* to enhance anti-inflammatory effects or miRNA-based therapies to correct dysregulated signaling pathways (Tarallo et al., 2022; Chen et al., 2022). These miRNAs target major pathways relevant to MDD, including axon guidance, circadian rhythm, and neurotrophin signaling. For example, miR-1976 shows a positive association with *Bifidobacterium*, targeting long-term potentiation pathways, whereas miR-1276 shows a negative association with *Collinsella*. This bidirectional interaction may indicate that host-derived miRNAs can modulate microbial compositions, and microbiota metabolites also regulate miRNA expression to impact gut integrity, inflammation, and brain function.

### 6.4 Mild cognitive impairment

Mild cognitive impairment (MCI), an early stage of Alzheimer’s disease (AD), is a condition in which individuals experience memory loss that exceeds what would be expected for their age (DeTure and Dickson, 2019). Dysbiosis of the gut microbiome has been implicated in numerous neurodegenerative disorders, including AD (He et al., 2020; Cryan et al., 2020). Some studies have indicated that alterations in the gut microbiome may potentially commence during the presymptomatic phase of AD, thereby contributing to its pathogenesis (Li et al., 2019; Nho et al., 2019). Diet, regarded as a modifiable risk factor for AD, exerts a substantial influence on the prevention of cognitive decline (Cryan et al., 2020).

A recent study conducted on middle-aged and elderly Chinese populations examined the association between diet quality, gut microbiome, and miRNA profiles in relation to MCI (Zhang et al., 2021). In this study with MCI revealed reduced microbial diversity with a significant decrease in the beneficial taxa *Faecalibacterium*, *unidentified Ruminococcaceae*, *Alistipes*, and *Rikenellaceae*, but increased pro-inflammatory taxa, including *Proteobacteria* and *Gammaproteobacteria*. These microbial changes are associated with the dysregulation of circulating miRNAs: decreased hsa-let-7 g-5p, hsa-miR-107, and hsa-miR-186-3p, but increased hsa-miR-144-3p. Noticeably, both *Proteobacteria* and *Gammaproteobacteria* are negatively correlated with hsa-let-7 g-5p, hsa-miR-107, and hsa-miR-186-3p, while microbial diversity is positively correlated only with hsa-miR-107 and hsa-miR-186-3p. This reciprocal interaction may suggest that dysbiosis might contribute to systemic inflammation and dysregulation of miRNA, which consequently modulates gene expression under pathways affecting microbial colonization and cognitive health (Zhang et al., 2021).

Furthermore, the study demonstrated that individuals with the highest CDGI-2018 (China Diet and Health Index) or HLS (Healthy Lifestyle Score) exhibited a 25 to 46% reduction in the likelihood of developing MCI. Conversely, elevated E-DII (dietary inflammatory index) scores were linked to a 46% heightened risk of MCI. These findings lend support to the notion that a diet and lifestyle conducive to good health are associated with a reduced risk of cognitive decline and the onset of AD (Dhana et al., 2020). Emerging evidence also suggests that neurotoxins, such as lipopolysaccharide (LPS) from the gut microbiome, exacerbate neuroinflammation in neurodegenerative diseases like AD. LPS promotes inflammatory pathways and miRNA upregulation, contributing to cognitive decline in AD. These miRNAs downregulate key immune-related genes, affecting synaptic function and immune responses, which are crucial in AD pathology (Lukiw and Pogue, 2020; Alexandrov et al., 2019; Zhan et al., 2016; Lukiw, 2020; Lukiw et al., 2019; Hill et al., 2015; Lukiw, 2016; Zhao et al., 2019; Lukiw et al., 2012; Zhao et al., 2016).

## 7 Current advances and therapeutic approaches: human milk and fecal microbiota transplantation

Human milk plays a critical role in shaping the infant’s gut microbiome and immune development. Maternal miRNAs, such as miR-378 and miR-320, transmitted through breast milk, help regulate microbial diversity and promote the growth of beneficial bacteria like *Bacteroides* and *Rothia*, which are linked to improved gut health and immune function. Moreover, maternal diet significantly influences these miRNA signatures, with plant-based diets enhancing the development of a healthy microbiome in the infant (Yeruva et al., 2023).

Fecal Microbiota Transplantation (FMT) effectively treats recurrent *Clostridioides difficile* (rCDI) and is being tested for dysbiosis-related conditions like IBD and metabolic disorders. FMT restores the microbiome’s diversity and has been shown to impact miRNA expression. In rCDI patients, FMT leads to the upregulation of miRNAs like miR-23a, miR-150, and miR-26b, which regulate inflammation and protect intestinal cells. These miRNAs target key cytokines involved in inflammatory diseases and autoimmunity, demonstrating that FMT not only restores microbial balance but also modulates molecular pathways to reduce inflammation. A recent study investigating FMT in subjects with metabolic syndrome revealed significant correlations between changes in microbiota composition and fecal miRNA profiles. For instance, *Blautia* and *Faecalibacterium* showed strong positive correlations with hsa-miR-2114-5p and hsa-miR-6833-5p, respectively, while *Odoribacter* and *Anaerostipes* correlated negatively with hsa-miR-3622b-5p and hsa-miR-3648-2-3p. These findings suggest that FMT-induced shifts in the microbiota can directly influence miRNA expression, potentially impacting host metabolic and inflammatory pathways. Interestingly, while the study did not find direct effects of specific miRNAs on bacterial growth *in vitro*, the observed correlations highlight the complex bidirectional communication between host miRNAs and gut microbiota (Wortelboer et al., 2022).

**Figure 2:**
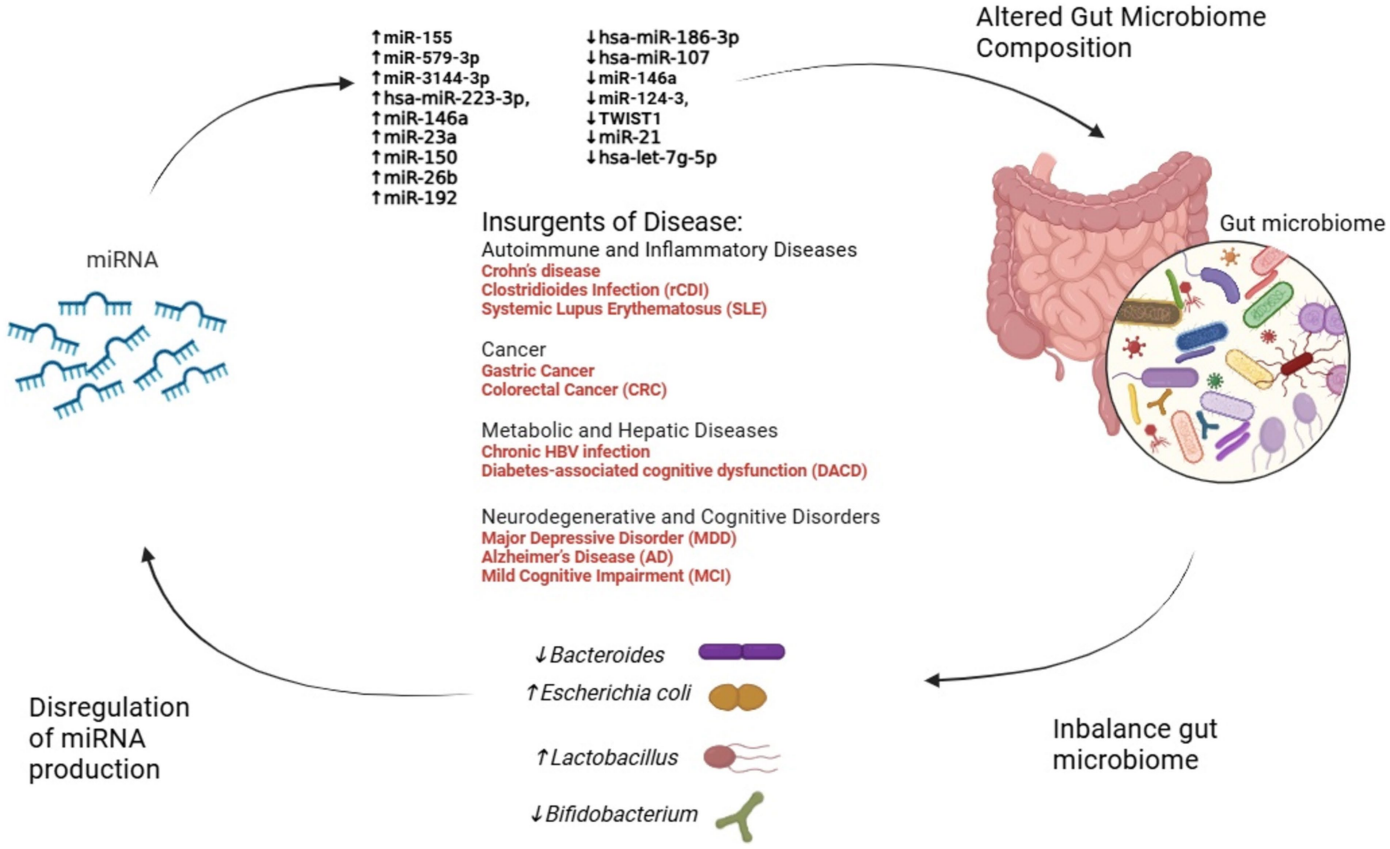
The interplay between miRNA dysregulation, gut microbiome alteration, and disease progression. This figure illustrates the dynamic interactions between miRNA dysregulation and gut microbiome composition across various diseases. Dysregulated miRNAs influence microbial balance, promoting shifts in key bacterial genera such as *Escherichia coli*, *Bacteroides*, *Lactobacillus*, and *Bifidobacterium*. In turn, these microbiome changes feedback to modulate miRNA expression, creating a reciprocal relationship. Disease categories—including autoimmune, cancer, metabolic, and neurodegenerative disorders—emerge from these alterations, emphasizing the critical role of the miRNA-microbiome axis in health and disease.

FMT also appears to restore miRNA biogenesis machinery, such as the enzyme DROSHA, which is suppressed during dysbiosis. This underscores the potential of FMT as a dual strategy: restoring microbial balance and correcting miRNA dysregulation (Monaghan et al., 2021). Figure 2 illustrates the intricate interplay between miRNAs and the microbiome.

## 8 Future perspectives for cardiovascular disease

**Figure 3:**
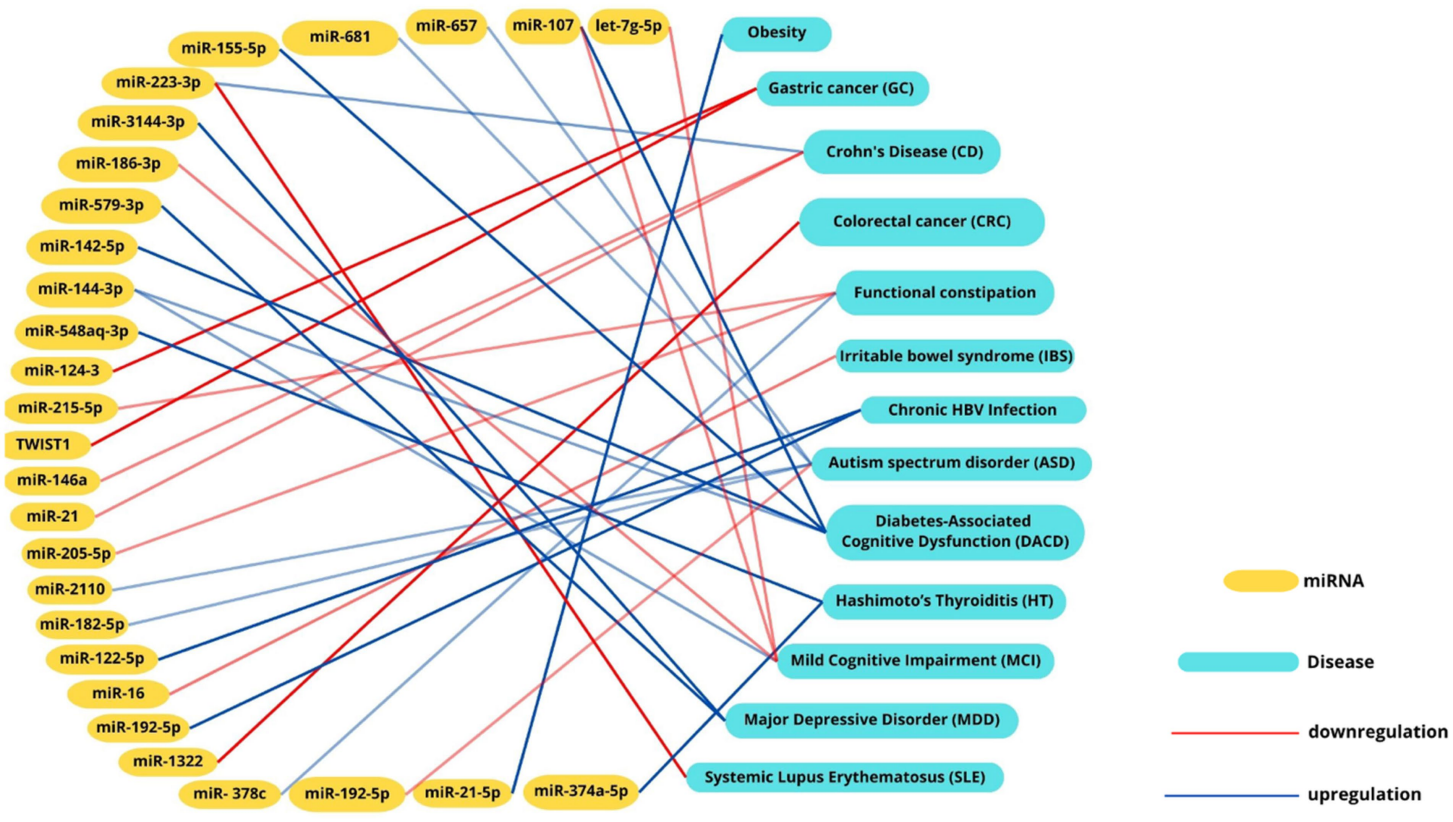
Intercation networks between miRNAs, microbial signatures and diseases. Interaction network illustrating the associations between miRNAs and their correlated diseases. Positive correlations are depicted with red lines, while negative correlations are represented by blue lines. This visualization highlights the intricate interplay between miRNA dysregulation and disease pathogenesis, emphasizing their potential roles in molecular mechanisms underlying various conditions.

Emerging research emphasizes the significant role of miRNAs as potential biomarkers in cardiovascular diseases (CVDs). For instance, miR-146a-5p’s association with the cardiometabolic risk factor trimethylamine-N-oxide (TMAO), a microbial metabolite linked to atherosclerosis. While studies in murine models that miR-146a-5p regulates key targets such as Numb and Dlst, which are inversely associated with LDL cholesterol levels and contribute to atherosclerosis progression (Coffey et al., 2019) further investigation in human studies is needed. Similarly, microbial metabolites like TMAO have been shown to modulate miRNA expression through inflammatory pathways such as NF-κB (Ionescu et al., 2022). The interplay between gut microbiota, miRNAs, and cardiovascular risk is further evidenced by microbial imbalances. Increases in TMA-producing bacteria, such as *Firmicutes* and *Proteobacteria*, alongside reductions in butyrate-producing microbes like *Bifidobacteria*, contribute to elevated TMAO levels. This, in turn, influences miRNA profiles, including the upregulation of pro-inflammatory miR-155 and suppression of protective miR-126, exacerbating endothelial dysfunction, inflammation, and plaque formation (Graham et al., 2023). These findings underline the bidirectional relationship between dysbiosis and miRNA regulation. Recent insights into gastrointestinal conditions, such as prolonged constipation, have further highlighted the interconnectedness of gut health and systemic diseases. Chronic constipation has been associated with an increased risk of colon cancer and cardiovascular or cerebrovascular events, potentially mediated by microbiota-driven dysbiosis and miRNA dysregulation. These findings emphasize the importance of considering gut health as a contributor to overall cardiovascular and metabolic risk (Power et al., 2013; Dong et al., 2023). Such observations have opened new avenues for research into the mechanisms linking gut health, miRNA expression, and systemic diseases. Future research should delve deeper into the molecular mechanisms underlying miRNA-microbiome interactions, particularly focusing on signaling pathways that regulate inflammation, endothelial function, and lipid metabolism. Understanding how miRNAs influence microbial metabolite production, such as TMAO and butyrate, as well as gut barrier function, could provide new insights into disease pathophysiology (Zhou et al., 2024). Similarly, studies should investigate how microbial communities reciprocally regulate miRNA biogenesis, potentially through the modulation of key enzymes like DROSHA and DICER (Treiber et al., 2019). Therapeutic miRNA-based approaches hold significant promise. Targeted delivery systems, such as nanoparticles or engineered exosomes, could be used to modulate specific miRNA profiles in cardiovascular tissues, restoring protective miRNAs like miR-126 or suppressing pro-inflammatory miRNAs like miR-155 (Gondaliya et al., 2024; Mahesh and Biswas, 2019; Rastegar-Moghaddam et al., 2023). Engineered probiotics, designed to produce beneficial metabolites or even miRNA mimics, represent another innovative strategy to correct dysbiosis and modulate gene expression. Such interventions could address the root causes of chronic inflammation and metabolic dysregulation in CVDs (Murali and Mansell, 2024; Jiang et al., 2024).By connecting these emerging insights to future therapeutic approaches, the integration of gut microbiota modulation and miRNA-targeted strategies represents a promising frontier for improving cardiovascular outcomes. The recognition of these intricate interconnections underscores the need for a multidisciplinary approach to uncover novel diagnostics and interventions (Figure 3).

## 9 Conclusion

This review highlights the intricate interplay between the gut microbiome and microRNAs across a wide spectrum of human diseases, including gastrointestinal disorders, autoimmune conditions, neurodegenerative diseases, and mental health disorders. The bidirectional influence of these elements represents a transformative area in precision medicine, offering opportunities for innovative diagnostics and therapeutics. Despite these advances, current research faces significant limitations, such as variability in methodologies, challenges in clinical translation, and difficulties in achieving targeted delivery mechanisms for miRNA-based therapies. Ethical considerations also emerge, with concerns about off-target effects, microbial imbalances, and potential transgenerational impacts of microbiome alterations. Addressing these issues requires robust ethical frameworks emphasizing transparency, informed consent, and long-term monitoring. Moreover, regulatory approval frameworks must include rigorous preclinical validation, phased clinical trials, and post-market surveillance to ensure safety and efficacy. Future research should focus on elucidating the molecular mechanisms of miRNA-microbiome interactions, identifying biomarkers, and developing innovative therapeutic delivery systems, such as engineered probiotics or nanoparticle carriers, to modulate gene expression and restore microbial balance. Personalized medicine approaches integrating molecular, genetic, and microbiome data will be pivotal in addressing these challenges and advancing precision therapies. The transformative potential of miRNA and microbiome-targeted interventions is underscored by ongoing clinical trials (see Table 2). These trials highlight promising therapeutic approaches, emphasizing the importance of translating research findings into clinical applications that improve patient outcomes. By addressing current limitations and fostering interdisciplinary collaboration, the miRNA-microbiome axis offers a pathway to develop safer, more effective, and personalized solutions for chronic diseases, paving the way for significant advancements in precision medicine.

**Table 2:** Clinical trials involving miRNA-based therapeutics and microbiome interventions.

Trial ID	Phase	Condition	Intervention	Key remarks
NCT03680274	Phase 1/2	Autism Spectrum Disorder	Fecal Microbiota Transplantation (FMT)	Investigating the safety and efficacy of FMT in children with ASD.
NCT01462734	N/A	Macular Hole	MicroRNA Expression Profiling	Studying microRNA expression in vitreous samples of patients with macular hole.
NCT00806650	N/A	Kidney Cancer	MicroRNA Signature Blood Test	Evaluating microRNA signatures in blood tests for detecting metastasis in kidney cancer patients.
NCT03294122	N/A	Prostate and Head–Neck Cancer	Microbiota and Inflammatory Markers Study	Assessing the role of microbiota and inflammatory markers in radiation-induced toxicity.
NCT04759625	N/A	Healthy Older Adults	Dietary Intervention with Mushroom-Enriched Biscuits	Examining the effect of β-glucan-rich biscuits on intestinal health and microbiota composition.

This table provides an overview of clinical trials investigating miRNA-based therapies and microbiome interventions. It includes trial identifiers, phases, conditions studied, interventions tested, and key objectives or findings, such as the use of fecal microbiota transplantation in autism, microRNA profiling in macular hole, and dietary modulation of gut microbiota in older adults.

## Data Availability

The original contributions presented in the study are included in the article/Supplementary material, further inquiries can be directed to the corresponding authors.
