# Correction: Systematic review of bidirectional interaction between gut microbiome, miRNAs, and human pathologies

**DOI:** 10.3389/fmicb.2026.1872999

**Published:** 2026-05-13

**Authors:** Lorenzo Drago, Luigi Regenburgh De La Motte, Loredana Deflorio, Delia Francesca Sansico, Michela Salvatici, Emanuele Micaglio, Manuele Biazzo, Fabiana Giarritiello

**Affiliations:** 1 UOC Laboratory of Clinical Medicine with Specialized Areas, IRCCS MultiMedica, Milan, Italy; 2 Clinical Microbiology and Microbiome Laboratory, Department of Biomedical Sciences for Health, University of Milan, Milan, Italy; 3 Genetic Consultant, IRCCS Multimedica, Milan, Italy; 4 The BioArte Ltd., Life Science Park, San Gwann, Malta; 5 Department of Medicine and Health Sciences “V. Tiberio”, University of Molise, Campobasso, Italy

**Keywords:** miRNA, gut microbioma, inflammatory bowel disease (IBD), Crohn's disease (CD), Alzheimer's disease (AD), major depressive disorder (MDD), cardiovascular disease

Incorrect **Funding**

In the published article, there was an error in the **Funding** statement. The original statement incorrectly read: “The author(s) declare that no financial support was received for the research, authorship, and/or publication of this article.”

The correct **Funding** statement is as follows: “The author(s) declared that financial support was received for this work and/or its publication. This work has been supported by the Ministry of Health—Ricerca Corrente to IRCCS MultiMedica.”

The original article has been updated.

